# Potential Biological Properties of Lycopene in a Self-Emulsifying Drug Delivery System

**DOI:** 10.3390/molecules28031219

**Published:** 2023-01-26

**Authors:** Sônia Nair Báo, Manuela Machado, Ana Luisa Da Silva, Adma Melo, Sara Cunha, Sérgio S. Sousa, Ana Rita Malheiro, Rui Fernandes, José Roberto S. A. Leite, Andreanne G. Vasconcelos, João Relvas, Manuela Pintado

**Affiliations:** 1 Laboratório de Microscopia e Microanálise, Departamento de Biologia Celular, Instituto de Ciências Biológicas, Universidade de Brasília, Campus Universitário Darcy Ribeiro, Asa Norte, Brasília 70910-900, DF, Brazil; 2 CBQF—Centro de Biotecnologia e Química Fina—Laboratório Associado, Escola Superior de Biotecnologia, Universidade Católica Portuguesa, Rua Diogo Botelho 1327, 4169-005 Porto, Portugal; 3 i3S—Instituto de Investigação e Inovação em Saúde, Universidade do Porto, 4200-135 Porto, Portugal; 4 Núcleo de Pesquisa em Morfologia e Imunologia Aplicada, Área de Morfologia, Faculdade de Medicina, Universidade de Brasília, UnB, Campus Universitário Darcy Ribeiro, Asa Norte, Brasília 70910-900, DF, Brazil; 5 People & Science Pesquisa, Desenvolvimento e Inovação Ltda, Brasília 70910-900, DF, Brazil

**Keywords:** nanomedicine, skin care, carotenoid, antioxidant, guava fruit

## Abstract

In recent years, lycopene has been highlighted due to its antioxidant and anti-inflammatory properties, associated with a beneficial effect on human health. The aim of this study was to advance the studies of antioxidant and anti-inflammatory mechanisms on human keratinocytes cells (HaCaT) of a self-emulsifying drug delivery system (SEDDS) loaded with lycopene purified from red guava (nanoLPG). The characteristics of nanoLPG were a hydrodynamic diameter of 205 nm, a polydispersity index of 0.21 and a zeta potential of −20.57, providing physical stability for the nanosystem. NanoLPG demonstrated antioxidant capacity, as shown using the ORAC methodology, and prevented DNA degradation (DNA agarose). Proinflammatory activity was evaluated by quantifying the cytokines TNF-α, IL-6 and IL-8, with only IL-8 showing a significant increase (*p* < 0.0001). NanoLPG showed greater inhibition of the tyrosinase and elastase enzymes, involved in the skin aging process, compared to purified lycopene (LPG). In vitro treatment for 24 h with 5.0 µg/mL of nanoLPG did not affect the viability of HaCaT cells. The ultrastructure of HaCaT cells demonstrated the maintenance of morphology. This contrasts with endoplasmic reticulum stresses and autophagic vacuoles when treated with LPG after stimulation or not with LPS. Therefore, the use of lycopene in a nanoemulsion may be beneficial in strategies and products associated with skin health.

## 1. Introduction

The use of biodiversity to search for bioactive compounds has gained increasing importance, enabling the development of effective, accessible and safe alternatives for the prevention and treatment of various conditions that affect human health [1,2]. Related to this context, the development of nanotechnology has represented a leap in science, bringing benefits such as physical-chemical stabilization of bioactive molecules, improved solubility and bioavailability, decreased toxicity, as well as potentiation of biological activities, providing applications in the pharmaceutical, food and cosmetics industries [3,4,5]. Among the nanotechnologies, the production and use of nanocarriers stand out as systems to transport bioactive molecules with applications in cosmetics, involving compounds of lipids, polymers or inorganic materials [6]. In this line, skin health has taken on great importance, mainly associated with ultraviolet radiation (UV), which is responsible for the greatest damage caused to the skin: when penetrating the epidermis and dermis, the rays generate reactive oxygen species (ROS), which can cause genetic mutations resulting from failures in the natural mechanisms of protection against ultraviolet radiation [7]. Currently, antioxidants from natural products have been sought for topical use in order to reduce oxidative damage. Some oils and plant extracts have proven antioxidant potential, as they prevent biological oxidation reactions and reduce the formation of free radicals [8]. The use of these active substances at the nanometer scale optimizes their properties and especially promotes their penetration to the basal layer of the epidermis. There, they can indirectly help in the skin’s natural protection mechanisms, fighting free radicals, preventing severe cell damage and improving the general conditions of the skin [9,10]. Thus, guava (*Psidium guava* L.), a plant with a history of medicinal application, has received attention, as its fruit is rich in vitamins A and C, iron, phosphorus, calcium and minerals, complemented by a high level of organic and inorganic compounds, including antioxidant secondary metabolites [11]. Among these, lycopene, from the carotenoid family, acts as an antioxidant against the oxidation of proteins, lipids and DNA, improving the cellular antioxidant defense system [12,13,14]. From this perspective, the use of the biotechnological potential of red guava is envisaged, by means of a more concentrated extract of lycopene purified from red guava (LPG) [15]. LPG presents lycopene mainly in the cis isoform (5-Z-lycopene) and its antioxidant, antimicrobial and anti-inflammatory activities were previously reported [15,16]. However, biotechnological applications of lycopene are limited by its instability regarding temperature, oxygen and light, and poor water solubility [8,13]. In this context, a self-emulsifying drug delivery system (SEDDS) loaded with lycopene purified from red guava, named nanoLPG, was developed [17]. NanoLPG was able to stabilize lycopene at 5 °C for 10 months, while free LPG degraded in the first month of storage. It exhibited radical scavenging activity, as shown by ABTS method, and cytotoxic activity against human prostate cancer cells [17]. Now, the present proposal aims to use nanoLPG to advance knowledge of its antioxidant and anti-inflammatory potential, and to evaluate actions on the cell morphology of the human keratinocyte-lineage HaCaT.

## 2. Results and Discussion

### 2.1. Characterization of the Nanoemulsion—NanoLPG

Lycopene purified from red guava (LPG) was previously characterized by Amorim et al. [15] and Vasconcelos et al. [17]. The parameters of the absorption spectrum determined by the authors corresponded to lycopene 5-Z and a yield of >90% of lycopene per dry extract weight. Self-emulsifying drug delivery systems loaded with lycopene purified from red guava (nanoLPG) were produced according to Vasconcelos et al. [17], using sorbitan monostearate, polysorbate 80 and coconut oil. Sorbitan monostearate and polysorbate 80 are non-ionic surfactants that are often used as emulsifiers in the food and cosmetics industries, and in nanostructured systems. They are practically non-toxic [17,18,19,20] and have a relatively high hydrophilic–lipophilic balance (HLB), which is important for rapid formation of o/w droplets and good self-emulsifying performance [21]. A cold-pressed virgin natural coconut oil was used to solubilize LPG and as a source of medium-chain triglycerides (fatty acids between C6 and C20) to form a hydrocarbon network that contributes to the retention of lycopene in the system [19]. In this formulation, LPG is the bioactive component responsible for the antioxidant and beneficial properties to health are well described in the literature [15,16,17].

The physical-chemical characterization of nanoLPG was consistent with that previously described for the same sample [17]. DLS analysis results indicated that nanoLPG is composed of polydisperse droplets with an average diameter of approximately 205.73 nm (Table 1). Nanoparticle size can affect the stability of the delivery system by processes such as sedimentation or aggregation, as well as cellular internalization and toxicity; and for this reason, it is an important parameter for biological applications [22,23]. Therefore, in this study, nanoparticle size was similar to that reported in previous works on lycopene nanoparticles (211.30 ± 2.5, 250 ± 8.21, and 239.90 ± 8.67 nm) [17,19]. Polydispersity index (PdI) data (Table 1) indicated the nanoparticle-sized distribution of polydisperse droplets which is adequately uniform, because PdI values from 0.1 to 0.5 express a homogeneous size distribution [24]. The zeta potential represents the charge on the surface of nanoparticles [25]. The zeta potential of nanoLPG indicated an anionic character (Table 1), contributing to the electrostatic repulsion, prevention of aggregation and physical stability of the nanosystem.

### 2.2. Antioxidant Capacity

The antioxidant capacity of LPG and nanoLPG was analyzed using the ORAC methodology. Table 2 shows the results obtained, indicating a clear difference between the antioxidant activity of nanoLPG and that of LPG.

The results demonstrated a significant improvement (ca. 6 fold) of the antioxidant activity of lycopene when engineered into a nanoemulsion based on sorbitan manostearate and coconut oil. NanoLPG showed ORAC results of 2924.68 ± 234.80 µmol TE (Trolox equivalent)/mL, while free lycopene (LPG) showed ORAC results of 515.14 ± 55.30 µmol TE (Trolox equivalent)/mL. In a previous study, Amorim et al. [15] evaluated the antioxidant activity for lycopene extract (LEG) and purified lycopene (LPG) by the ORAC method, and the results obtained were 402.80 ± 44.40 and 315.39 ± μMol Trolox g^−1^, respectively.

The value obtained in this study for the antioxidant activity differs from the results observed in a lycopene nanoemulsion (127.66 ± 0.45 mM Trolox g^−1^) in mineral oil, through the ABTS assay, in which the antioxidant action of the pure extract (139.52 ± 0.30 mM Trolox g-1) is slightly higher [13]. Therefore, the results suggest that the antioxidant activity of lycopene from red guava may also be related to the composition of the nanoemulsion, and the size of the particles may also be as reported by Ha and co-workers, studying nanoemulsions of different sizes for tomato extract enriched with lycopene [26].

### 2.3. Agarose Gel Electrophoresis for DNA Protection

Since ORAC assay results hinted at a significant antioxidant capacity, the next step was to analyze whether LPG and nanoLPG were capable of preventing DNA degradation, as lycopene compounds can both be good protectors of DNA oxidation and also have pro-oxidant activity [8,27,28].

**Figure 1:**
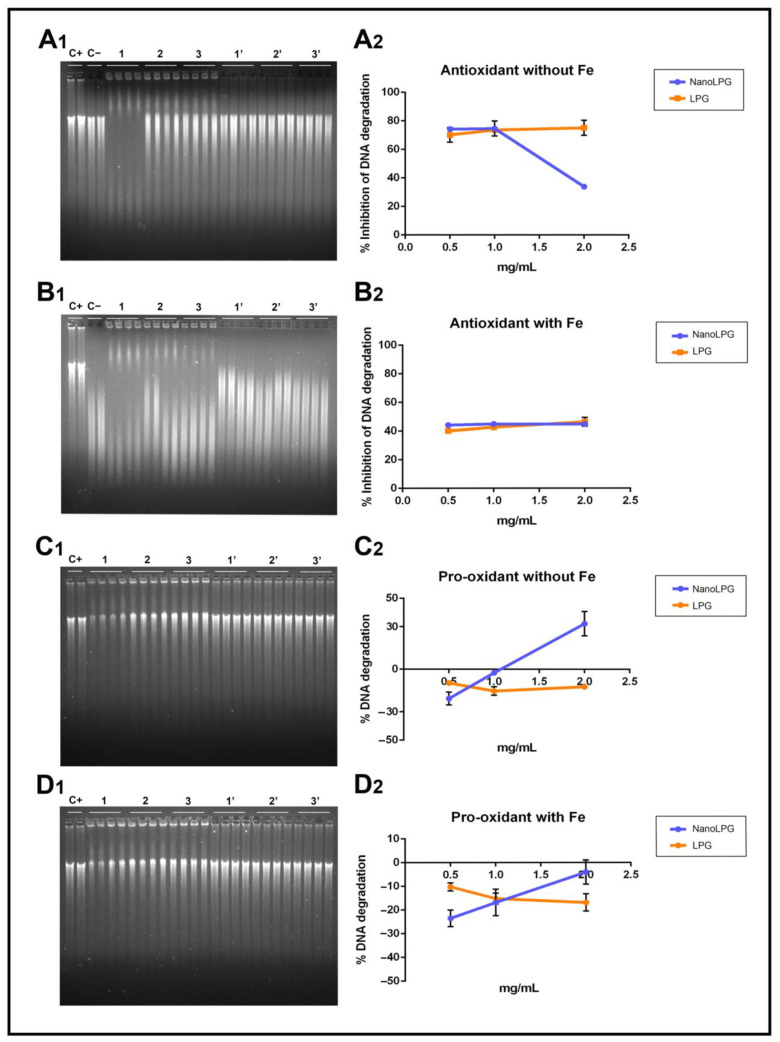
Electrophoresis results and the corresponding graphic of antioxidant activity in the prevention of DNA oxidation by H_2_O_2_ (**A_1_**,**A_2_**) and H_2_O_2_/FeCl_3_ (**B_1_**,**B_2_**) and the pro-oxidant effect in the absence (**C_1_**,**C_2_**) and presence (**D_1_**,**D_2_**) of iron cations of the different concentrations (0.5, 1 and 2 mg/mL (*v*/*v*)) of nanoLPG and LPG). (C+) DNA solution; (C−) DNA solution + degradation system; (1, 2 and 3) nanoLPG at 2, 1 and 0.5 mg/mL (*v*/*v*), respectively; (1′, 2′ and 3′) LPG at 2, 1 and 0.5 mg/mL (*v*/*v*), respectively. The data represent the mean ± SD from one experiment.

Both samples, in the presence of H_2_O_2_, resulted in ca. 70% of inhibition of DNA degradation, with the exception of the highest concentration (2 mg/mL) of nanoLPG, where the protection was in the range of 30% (Figure 1A_1_,A_2_). This means that all concentrations of LPG and nanoLPG were able to inhibit, even partially, the DNA cleavage induced by H_2_O_2_ peroxide, which is an oxidative process. This interaction is supported by the results of the pro-oxidant assay (Figure 1C_1_,C_2_), where the percentage of DNA degradation is negative, in the absence of iron cations when incubated with LPG and at lower concentrations (0.5 and 1 *v*/*v*) of nanoLPG. However, in the presence of iron, there was no cleavage of DNA subjected to LPG and nanoLPG (Figure 1D_1_,D_2_). Additionally, LPG and nanoLPG can interact with DNA, or open the strand, thus intensifying fluorescence, justifying the negative values. Similar results were described in studies using extracts from brewer’s spent grain (BSG) [29]. In the case of the highest concentration of nanoLPG (2 mg/mL), the inhibition was lower (Figure 1A_1_,A_2_). The highest concentrations of nanoLPG (1 and 2 mg/mL) (Figure 1A_1_,A_2_), instead of having a protective action on DNA, demonstrated a slight pro-oxidant effect, which leads to the recommendation to use low concentrations of nanoLPG. Such behavior is not observed when the system has iron (Figure 1B_1_,B_2_), since it probably complexes with iron ions at high concentrations, causing a reducing effect.

### 2.4. Enzyme Inhibition Assay

Tyrosinase and neutrophil elastase are enzymes involved in the skin aging process, with different effects. Tyrosinase is involved in melanin biosynthesis, with effects on skin browning. So, the use of tyrosinase inhibitors in cosmetic products may be helpful for preventing or reducing brown spots on the skin, which often occur with the aging process. Neutrophil elastase is involved in the degradation of several extracellular matrix proteins, such as collagen and elastin, leading to the loss of skin elasticity and firmness [30,31]. With aging, extracellular matrix compounds are reduced, leading to some aging-associated signs, such as spots and the loss of elasticity. So, molecules with the ability to act on these enzymes may have great commercial potential [32]. Thus, the samples’ ability to inhibit these two enzymes was tested (Table 3).

NanoLPG showed the best results for both enzymes, with the highest difference being observed for elastase. Compared to extracts described in other studies, nanoLPG and LPG may be promising as elastase and tyrosinase inhibitors. Nannochloropsis extracts inhibited 59.2 and 24.9% of tyrosinase and elastase activity, respectively, at 1000 µg/mL [33]; ethanol extracts from the tunicate *Styela clava* inhibited 100% of elastase activity, at 10,000 µg/mL [34]; tomato extracts inhibited 73.12 and 41.16% of elastase and tyrosinase, respectively, at 100 µg/mL [35]. Some extracts found in the literature showed higher inhibitory activity than nanoLPG and LPG; however, the concentrations tested were much higher than ours. So, both samples may be further studied regarding their cosmetic applications, with a greater prominence of nanoLPG.

### 2.5. Cytotoxicity

**Figure 2:**
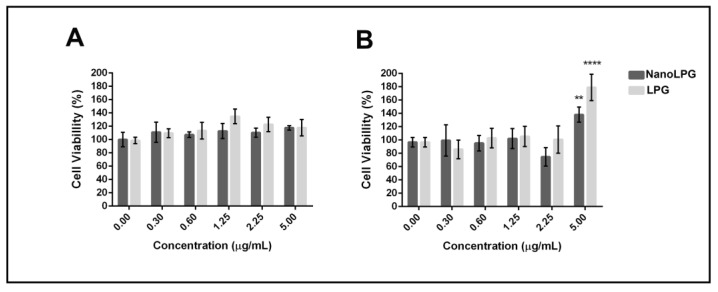
Cytotoxicity of free LPG and nanoLPG after 24 h. (**A**) HaCaT and (**B**) L929 (NCTC) cell viability. Bars represent cell viability, measured by the PrestoBlue assay (ThermoFisher), in percentage after treatments at the indicated concentrations. The data represent the mean ± SD of one independent experiment in quintuplicate. ** *p* < 0.01 and **** *p* < 0.0001. Treatment compared to untreated control.

We advanced the study to evaluate the cytotoxicity of the nanoemulsion on the human keratinocyte (HaCaT) and mouse fibroblast cell line L929 (NCTC). The PrestoBlue assay demonstrated that the LPG and nanoLPG treatments did not significantly affect the cell viability of both cell lines after 24 h exposure, even at the highest concentration used (5 µg/mL) (Figure 2).

These results contrast with those obtained previously, using the same formulation (nanoLPG), at a lower concentration (3.125 µg/mL), in the DU-145 prostate tumor cell line, while nanoLPG was not significantly cytotoxic on human peripheral blood mononuclear cells at 25 µg/mL [17]. Additionally, lycopene in the nanostructure formulation, such as the use of fucan-coated acetylated cashew gum nanoparticles (NFGa), showed significant cytotoxicity against MCF-7 from the lowest concentration (6.25 µg/mL), but did not affect cell viability from HaCaTC [8]. These results suggest that the action of lycopene is dependent on the formulation established and used in the assays, as well as on the cellular model. Therefore, this enables the use of lycopene in a nanoemulsion, which allows solutions for their chemical instability and low bioavailability and consequent use of their antioxidant and antitumor potential [12,36,37,38].

The significant difference observed in the viability of fibroblasts, lineage L929, suggests that at a dose of 5.0 µg/mL nanoLPG and LPG can accelerate the cell cycle. In vitro biocompatibility tests showed the significant cell adhesion, proliferation and viability of mouse L929 fibroblast cells using a poly(glyceril sebacate urethane) (PGSU) library containing different concentrations of hydroxyapatite (nHA) and curcumin nanoparticles [39]. According to Monika et al. [40], factors that interfere with the expression of vimentin and alpha-actin trigger changes in the cell cycle. In addition, studies using biomulti-functional thermibenzaldehyde, carboxymethyl chitosan and basic fibroblast growth factor (hydrogels (BP/CS-bFGF)) were prepared, and were shown to be effective for healing skin wounds [41]. In other in vitro studies, only keratocyte cells of human origin, HaCaT lineage, were used.

### 2.6. Inflammatory Response

**Figure 3:**
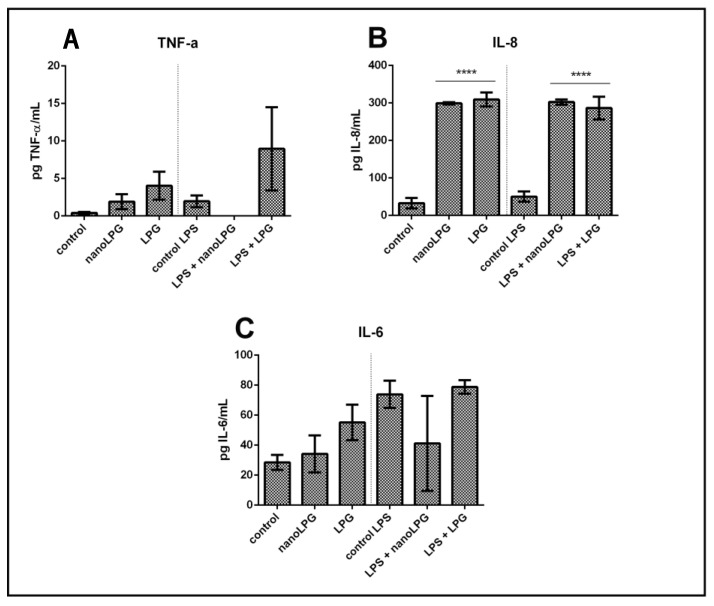
Action of free LPG and nanoLPG on the production of TNF-α (**A**), IL-6 (**B**) and IL-8 (**C**) in HaCaT cells. The left part of all graphs corresponds to the non-stimulated cell response, and the right is related to the anti-inflammatory effect. The production of TNF-α, IL-6 and IL-8 was determined by ELISA. The data represent the mean ± SD of one independent experiment in duplicate. **** *p* < 0.0001. Treatment compared to control and control LPS.

In order to verify the action of free lycopene (LPG) and LPG in a nanoemulsion (nanoLPG) on anti-inflammatory activity, HaCaT keratinocytes were stimulated by lipopolysaccharides (LPS), an inflammation-inducing agent. These cells were subjected to treatment at a concentration of 5.0 µg/mL with nanoLPG or LPG (Figure 3).

Immunomodulatory activity was evaluated in HaCaT cells by quantifying the pro-inflammatory cytokines TNF-α, IL-6 and IL8. The presence of LPG and nanoLPG resulted in different effects. The production of IL-8 significantly increased in the presence of LPG and nanoLPG samples, when compared to the control (Figure 3C). This result was observed in both conditions in the absence and presence of the stimulus with LPS. On the other hand, in relation to IL-6 (Figure 3B) and TNF-α (Figure 3A), although no significant differences were observed (*p* > 0.05) when compared to the control, the samples tested show a pro-inflammatory profile in the presence and absence of LPS. Therefore, it was observed that a concentration of 5.0 µg/mL was not cytotoxic in the Prestoblue cell viability assay (Figure 1) but exhibited anti-inflammatory potential. The presented results suggest an anti-inflammatory effect of the nanoemulsion only for TNF-α; and pro-inflammatory for IL-6 and IL-8, since the observed values are above the control values, that is, above the baseline value.

Lycopene has been shown to have an anti-inflammatory effect [42]. Studies reported by Moia et al. [14] also show that formulated nanolycopene has superior efficacy when compared to free (non-formulated) lycopene in animals with rheumatoid arthritis, which reinforces its action as an anti-inflammatory agent. In the present study, it was demonstrated that lycopene, both free lycopene from red guava and LPG in a nanoemulsion, led to an increase in IL-8 production compared to controls (Figure 3C). The literature contains studies with prostate [43], ovarian [44] and colorectal [45] cancers that demonstrate cell proliferation and migration, as well as an inflammatory condition stimulated by increases in the production of the cytokine IL-8. However, studies have shown that glycolic acid, present in fruits, can decrease skin inflammation resulting from exposure to ultraviolet solar irradiation, as a result of decreased expression of IL-8 genes [46]. Machado et al. [47] recently demonstrated that the immune response is modulated in Caco-2 cells in the presence of pomegranate oil (PO), with a significant reduction in the secretion of IL-6 and IL-8 in unstimulated cells, as well as a significant reduction in all cytokines (TNF-α, IL-6 and Il-8) in stimulated cells. Therefore, it is possible to understand that the molecules TNF-α, IL-6 and IL-8 participate in inflammatory responses [48].

### 2.7. Cell Morphology

**Figure 4:**
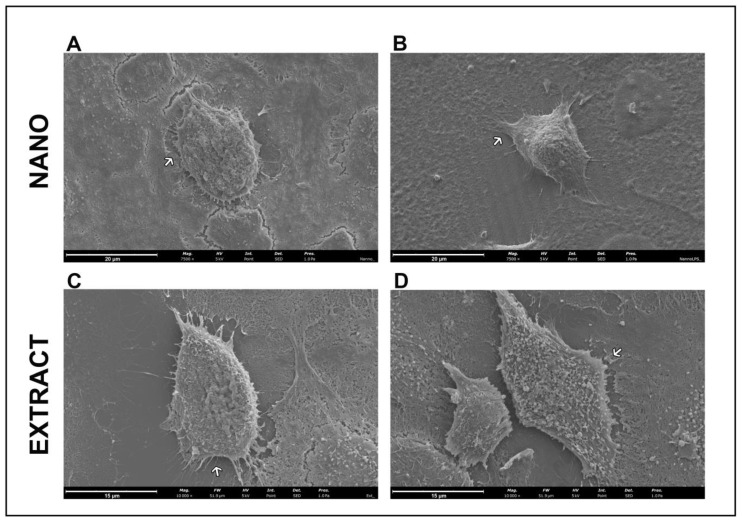
Scanning electron microscopy of HaCaT cells after 24 h of treatment with 5 µg/mL of nanoLPG (**A**,**B**) or LPG (**C**,**D**), in the absence (**A**,**C**) or presence (**B**,**D**) of lipopolysaccharides (LPS) at 1 µg/mL. Arrows indicate extensions on the cell surface.

The evaluation by scanning electron microscopy showed, as can be seen in Figure 4, that the HaCaT cells maintained their morphology after nanoLPG (Figure 3A) and LPG (Figure 3C) treatments. Cells showed a fusiform shape with extensions on the cell surface. A similar morphology was described by atomic force microscopy in keratinocytes subjected to a lamellar liquid crystal system in studies evaluating the elasticity [49]. With the use of lipopolysaccharide (LPS), it is observed that no morphological alteration occurred in the cells treated with nanoLPG (Figure 3B). However, Figure 4D suggests reduced projections and the presence of numerous vesicles on the surface of HaCaT cells challenged with LPS and treated with LPG.

**Figure 5:**
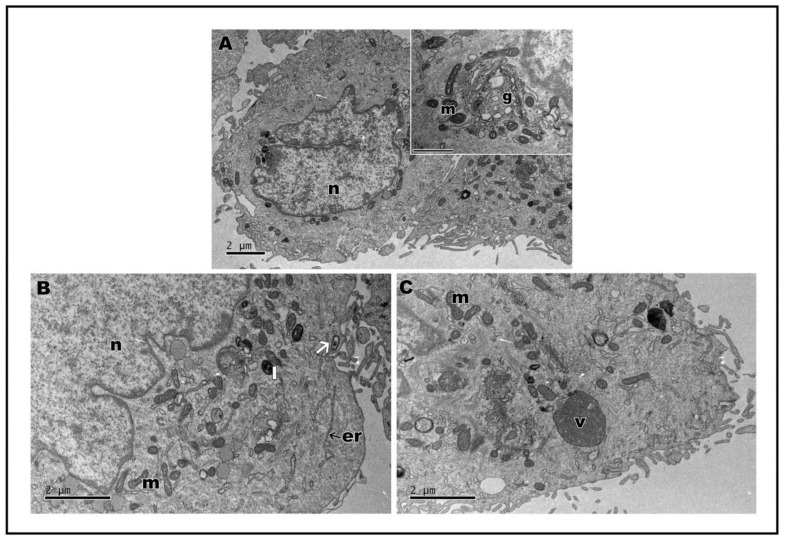
Transmission electron microscopy of HaCaT cells. Control cells (**A**); insect cytoplasmic region showing the Golgi complex. After treatment, for 24 h, with 5 µg/mL of nanoLPG (**B**) or LPG (**C**). (m) mitochondria, (n) nucleus, (g) Golgi complex, (er) endoplasmic reticulum, (l) lysosome, (v) autophagy vacuole, and (arrow) vesicle with nanoLPG.

In transmission electron microscopy, the ultrastructure of keratinocytes subjected to nanoLPG treatment is similar to that of untreated keratinocytes (control) (Figure 5A). Cells show expansions on the surface, mitochondria and the Golgi complex (Figure 5A—inset). Cells subjected to treatment with nanoLPG did not show any change in their morphology, and it is possible to observe the structure of the nanoemulsion inside the cells, especially in the context of endocytic vesicles and lysosomes (Figure 5B). When treated with the free LPG, the presence of structures involved in the endocytosis process is accentuated, mainly in autophagy structures (Figure 5C). The interaction of the nanoemulsion in different cell types is already present in the literature, mainly in the description of the use of the nanoemulsion as a drug carrier in cellular models of breast cancer [50,51]. TEM images also showed evidence of the interaction and internalization of nanoLPG into the endosome in human prostate cancer cells (DU-145) [17]. In the present study, it was possible to verify that nanoLPG increased the efficiency in the absorption of lycopene by keratinocytes, and thus the efficiency of its action as an antioxidant [8,13].

**Figure 6:**
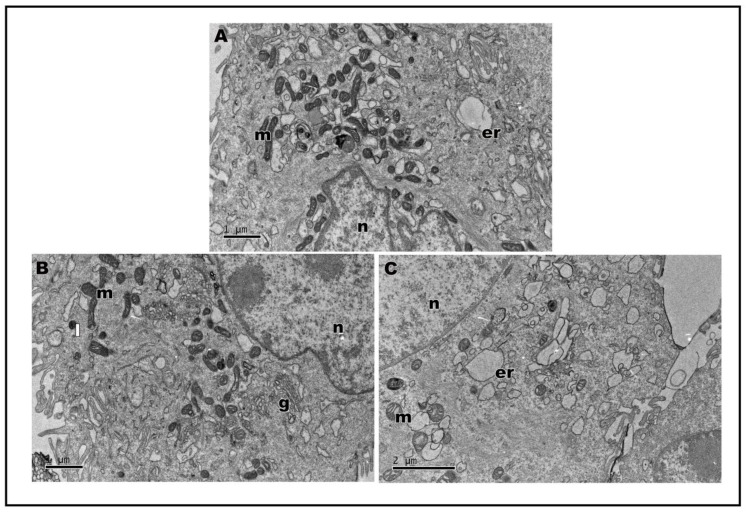
Transmission electron microscopy of HaCaT cells after 24 h of treatment with lipopolysaccharides (LPS) at 1 µg/mL (**A**). Cell treatment with 5 µg/mL of nanoLPG (**B**) or LPG (**C**) in the presence of lipopolysaccharides (LPS) at 1 µg/mL. (m) mitochondria, (n) nucleus), (g) Golgi complex, (er) endoplasmic reticulum, and (l) lysosome.

Assays were also carried out to analyze the ultrastructure of HaCat cells after stimulation with lipopolysaccharides (LPS) from Escherichia coli, a known inducer of inflammation, followed by treatment with nanoLPG and free LPG. LPS-stimulated keratinocytes demonstrated the cytoplasm with vacuoles (Figure 6A); when treated with nanoLPG, the morphology was observed (Figure 6B) to be similar to that of control cells not subjected to any treatment (Figure 5A). However, when subjected to LPG, it is possible to observe the vacuolated cytoplasm (Figure 6C). Changes in the ultrastructure of cytoplasmic organelles, notably in the endoplasmic reticulum, were noted, namely distension, which is a known manifestation of ER stress, and autophagic vesicles, which were observed in HaCaT cells treated with KL3, a podophytoxin compound with anticancer properties [52].

The literature also mentions changes in keratinocyte mitochondria resulting from the action of polluting components, which are capable of modulating signal transduction pathways, resulting in the activation of inflammatory processes in the skin, followed by oxidative damage [53].

## 3. Materials and Methods

### 3.1. Lycopene Extraction

Lycopene purified from red guava (LPG) was produced according to Amorim et al. [15]. Highly ripe red guava (*Psidium guajava* L.) from Parnaíba city, State of Piauí, Brazil, were subjected to dehydration with ethanol and extraction with dichloromethane under ultrasonic stirring. The extract was then filtered through quantitative filter paper and dried under reduced pressure (50 mbar) at room temperature in an R-215 rotary evaporator (Büchi Labortechnik, Flawil, Switzerland) under dim light. After that, purified lycopene was obtained by crystallization at −20 °C and washing with ethanol and chloroform. The isolated lycopene was stored at −80 °C.

### 3.2. Production of SEDDs Containing Lycopene Purified from Red Guava

Self-emulsifying drug delivery systems containing lycopene purified from red guava (nanoLPG) were produced according to Yen et al. [54], with modifications by Vasconcelos et al. [17]. LPG, sorbitan monostearate, and coconut oil (1:10:0.06, *w*:*w*:*v*) were mixed in ethanol:acetone (1:8, *v*:*v*) under magnetic stirring for 10 min at 40 °C. The mixture was poured into distilled water (pH 7.0) containing polysorbate 80 (0.45 g) under the same conditions of stirring and temperature, following the organic:aqueous phase proportion of 1:2.5 (*v*:*v*). The formulation was concentrated under reduced pressure (30 mbar) at 37 °C in an R-215 rotary evaporator (Büchi Labortechnik, Switzerland) until a final volume of 20 mL, eliminating the organic solvents. The nanoLPG formulation was stored in tightly closed plastic bottles in a refrigerator (5–8 °C). The preparation of nanoLPG was carried out under dim light.

### 3.3. NanoLPG Characterization

#### 3.3.1. Particle Size and Charge

NanoLPGs were diluted in water and analyzed in terms of their physical properties by dynamic light scattering (DLS) using a Malvern Instruments NanoZSP (Worcestershire, UK). The measured parameters were hydrodynamic diameter (HD), the polydispersity index (PdI) and the zeta potential (ZP). All assays were performed using a disposable folded capillary cell (Malvern, Panalytical, Worcestershire, UK), with a 90° laser angle and at room temperature (25 °C).

#### 3.3.2. Antioxidant Capacity

The total antioxidant activity for the nanoemulsion was determined by the oxygen radical absorbance capacity assay (ORAC-FL) method. The reaction was carried out in 75 mM phosphate buffer (pH 7.4), and the final reaction mixture was 200 µL. Antioxidant (20 µL) and fluorescein (120 µL; 70 nM, final concentration in well) solutions were placed in the well of the microplate. A blank (fluorescein + AAPH) using phosphate buffer instead of the antioxidant solution and eight calibration solutions using Trolox (1–8 µM, final concentration in well) as antioxidant were also carried out in each assay. The mixture was pre-incubated for 10 min at 37 °C. AAPH solution (60 µL; 12 mM, final concentration in well) was added rapidly. The microplate was immediately placed in the reader and the fluorescence was recorded at intervals of 1 min for 80 min. This assay was performed with a multi-detection plate reader (Synergy H1; BioTek Instruments, Winooski, VT, USA) controlled by the Gen5 Biotek software version 3.04. The excitation wavelength was set at 485 nm and the emission wavelength at 528 nm. The microplate was automatically shaken before each reading. Black polystyrene 96-well microplates (Nunc, Roskilde, Denmark) were used. AAPH and Trolox solutions were prepared daily and fluorescein was diluted from a stock solution (1.17 mM) in the same phosphate buffer. Antioxidant curves (fluorescence versus time) were first normalized to the curve of the blank corresponding to the same assay by multiplying original data by the factor fluorescence_blank,t = 0_/fluorescence_control,t = 0_. From the normalized curves, the area under the fluorescence decay curve (AUC) was calculated according to the trapezoidal method. The final AUC values were calculated by subtracting the AUC of the blank from all the results. Regression equations between the net AUC and antioxidant concentration were calculated. Final ORAC values were expressed as μmol TE (Trolox equivalent)/mL.

#### 3.3.3. Agarose Gel Electrophoresis for DNA Protection

The method was performed as previously described by Silva et al. [55]. Antioxidant and pro-oxidant assays were performed. The degradation systems applied in the antioxidant assay were hydrogen peroxide (H_2_O_2_) (Sigma, Merck KGaA, Darmstadt, Germany) 7.01 M and H_2_O_2_ with iron (III) chloride (Sigma, Merck KGaA, Darmstadt, Germany) (FeCl_3_) 10 mM. Various concentrations of LPG or nanoLPG were used (2, 1 and 0.5% (*v*/*v*)). Deoxyribonucleic acid sodium salt (DNA) (Sigma, Merck KGaA, Darmstadt, Germany) was incubated in the presence of the degradation systems, of nanoLPG or LPG. As a negative control, a solution of DNA without H_2_O_2_ was used for the assay with the H_2_O_2_ system, and a solution of DNA with 10 mM of FeCl_3_ for the assay using the H_2_O_2_/FeCl_3_ system. It was incubated for 1 h in the dark at 37 °C, and then agarose gel electrophoresis was run. In the pro-oxidant assay, the DNA was incubated with and without 10 mM FeCl_3_ in the presence of various concentrations of LPG or nanoLPG (2, 1, and 0.5% (*v*/*v*)). It was also incubated for 1 h in the dark at 37 °C. In the electrophoresis gel, each sample was mixed 1:4 with loading buffer (25 mg bromophenol blue, 10 mL Tris EDTA (TE) buffer 1x pH 8.0 (Sigma, Merck KGaA, Darmstadt, Germany) and 20 mL glycerol (Thermo Fisher Scientific, Walthem, MA, USA) (C_3_H_8_O_3_) with a pH value adjusted to 8.0) and aliquots of 10 mL were transferred into a 0.75% (*w*/*v*) agarose (Nztech, Lisbon, Portugal) gel prepared using a Tris-Acetate EDTA buffer (TAE) (BioRad, Hercules, CA, USA) supplemented with 0.03 µL/mL GreenSafe Premium (Nzytech, Lisbon, Portugal). The gel was run for 1.25 h at 150 mV. A molecular imager, GelDoc XR+ (BioRad, Hercules, CA, USA), was used to analyze the gels, and Image Lab Software v5.1 (BioRad, Hercules, CA, USA) was used to process the resulting image. The band intensity of the positive control was measured by manually defining the band area, and this area was copied into each sample lane. The decrease in the band intensity was considered as a result of the reduction in the amount of DNA present. The results are presented as a percentage of inhibition of the DNA band degradation (for the antioxidant assay) and as a percentage of DNA band degradation (for the pro-oxidant assay), both calculated using the equations below—Equations (1) and (2).

Inhibition of DNA degradation (%) = (Intensity_sample_ − Intensity_background_)/Intensity_DNA_ solution × 100 (1)

DNA degradation (%) = 100 − [(Intensity_sample_ − Intensity_background_)/Intensity_DNA_ solution × 100] (2)

where intensity_sample_ is the intensity of each sample band, intensity_background_ is the intensity of the background, measured beside the control bands, and intensity_DNA_ solution represents the intensity of the intact DNA solution. All incubations were performed in triplicate and loaded twice into the gel.

#### 3.3.4. Aging-Related Enzyme Inhibition Assay

LPG and nanoLPG were evaluated regarding their ability to inhibit two enzymes related to the skin aging process, namely neutrophil elastase and tyrosinase. Both samples were tested in duplicate, at a concentration of 5 µg/mL, and dilutions were performed in each assay’s specific buffer. A tyrosinase inhibitor screening kit (abcam, ab204715) and a neutrophil elastase inhibitor screening kit (abcam, ab118971) were used, and the assays were performed according to the manufacturer’s instructions. For neutrophil elastase, SPCK (10 µM for human leukocyte elastase) was used as a control inhibitor, and the fluorescence reaction was measured at an excitation/emission of 400/505 nm in a multi-detection plate reader (Synergy H1; BioTek Instruments, Winooski, VT, USA), in the kinetic mode for 30 min at 37 °C. For tyrosinase, kojic acid (0.75 µM) was used as an inhibitor control, and the absorbance was measured at 510 nm, in the kinetic mode for 60 min at 25 °C. The samples’ relative inhibition % for neutrophil elastase and tyrosinase was calculated using Equations (3) and (4), respectively.

Neutrophil elastase relative inhibition (%) = (ΔRFU _Sample_/ΔRFU _Enzyme control_) × 100 (3)

Tyrosinase relative inhibition (%) = [(Slope _Enzyme control_ − Slope _Sample_)/Slope of EC] × 100 (4)

### 3.4. In Vitro Studies

#### 3.4.1. Cell Line Growth Conditions

The human keratinocyte cell line (HaCaT) was obtained from Cell Line Services (Oppenheim, Denmark) and were cultured, as well as, the mouse fibroblast cells, L929 (NCTC) (ECACC 85103115), at 37 °C in a humidified atmosphere of 95% air and 5% CO_2_, as monolayers using Dulbecco’s Modified Eagle’s Medium (DMEM) with 4.5 g/L glucose, L-glutamine without pyruvate (Lonza, Verviers, Belgium) containing 10% (*v*/*v*) fetal bovine serum (FBS, Biowest, Nuaillé, France) and 1% (*v*/*v*) Penicillin-Streptomycin-Fungizone (Lonza, Verviers, Belgium).

#### 3.4.2. Cytotoxicity Assay

The cytotoxicity of LPG and nanoLPG on HaCaT and L929 cells was evaluated using a PrestoBlue assay (Thermo Fischer, Waltham, MA, USA), according to the manufacturer’s instructions. The cells were seeded, at 1 × 10^4^ cells/well, in 96-well plates; and after 24 h, cells were exposed to the samples at different concentrations (5–0.3 µg/mL) for 24 h in quintuplicates. Cells treated with 10% DMSO were used as a negative control. After incubation, PrestoBlue reagent was added to the medium and it was incubated for 1 h. The fluorescence signal was read in a Synergy H1 microplate reader (BioTek, Winooski, VT, USA). The results are expressed as the percentage of metabolic inhibition as compared with the control (cells without treatment).

#### 3.4.3. Anti-Inflammatory Capacity

HaCaT cells were seeded at 2.5 × 10^5^ cells/well in a 24-well microplate and incubated for 24 h. After 24 h, the culture medium was replaced with a medium supplemented with 5.0 µg/mL of LPG or nanoLPG in the presence or absence of lipopolysaccharides from Escherichia coli O111:B4 (LPS, Invitrogen) at 1 µg/mL to induce inflammation, and the plate was re-incubated for another 24 h. Interleukin 6 (IL-6) and 8 (IL-8) and Tumor Necrosis Factor-alpha (TNF-α) detection was performed by ELISA kits (Biolegend, San Diego, CA, USA) according to the manufacturers’ instructions.

#### 3.4.4. Scanning Electron Microscopy (SEM)

To visualize possible changes in cell shape and surface after treatments, cells were processed and analyzed using scanning electron microscopy (SEM). HaCaT cells were seeded at 2.5 × 10^5^ cells/well in a 24-well microplate with coverslips (Corning, New York, NY, USA) and incubated for 24 h. After 24 h, the culture medium was replaced with a medium supplemented with 5 µg/mL of LPG or nanoLPG in the presence or absence of lipopolysaccharides from Escherichia coli O111:B4 (LPS, Invitrogen) at 1 µg/mL to induce inflammation, and the plate was re-incubated for another 24 h. Fixation of the cells adhered to the coverslips was performed after medium removal, by using 2.5% (*v*/*v*) glutaraldehyde (Sigma-Aldrich, Saint Louis, MO, USA) solution for at least 1 h. Next, glutaraldehyde solution was removed and cells were washed by immersing the coverslips in deionized water (for 10 min), and then they were dehydrated by a graded ethanol series (30, 50, 70, 80, 90 and 100% (*v*/*v*)) also through immersion in each solution for 10 min. After removing the 100% ethanol solution, a couple of drops of hexamethyldisilazane (HMDS; Sigma-Aldrich, Saint Louis, MO, USA) were placed over the cells and immediately evaporated with a gentle stream of nitrogen. Coverslips were placed on top of observation pins (covered with double-sided adhesive carbon tape-NEM tape; Nisshin, Chiyoda-ku, Tokyo, Japan), and sputter coated with gold/palladium. The observation was performed in a scanning electron microscope (SEM), Phenom XL G2 by Thermo Fischer Scientific-FEI, Waltham, MA, USA (Eindhoven, Noord-Brabant, Netherlands) with a secondary electron detector (SED).

#### 3.4.5. Transmission Electron Microscopy (TEM)

To visualize possible changes in cell structures after treatments, cells were processed and analyzed using transmission electron microscopy (TEM). HaCaT cells at a density of 1 × 10^6^ cell/well were treated or not (control) with LPG or nanoLPG (5.0 µg/mL) and incubated at 37 °C for 24 h. In another assay after 24 h, the culture medium was replaced with a medium supplemented with 5.0 µg/mL of LPG or nanoLPG in the presence or absence of lipopolysaccharides from Escherichia coli O111:B4 (LPS, Invitrogen) at 1 µg/mL to induce inflammation, and the plate was re-incubated for another 24 h. Then, the cells were detached with trypsin, centrifuged at 2500 rpm for 5 min at 4 °C, washed with PBS three times and fixed in 2.5% glutaraldehyde/2% paraformaldehyde in sodium cacodylate buffer 0.1 M (pH 7.4). Cells were then scraped and collected. Samples were washed in 0.1 M sodium cacodylate buffer, post-fixed in 2% osmium tetroxide in 0.1 M sodium cacodylate buffer overnight, and incubated in 1% uranyl acetate overnight, to improve membrane contrast. Dehydration was performed in gradient series of ethanol solutions and propylene oxide. Samples were included in Epon resin by immersion in increasing series of propylene oxide and EPON (until 0:1 ratio) for 60 min each. Sections with a thickness of 60 nm were prepared on RMC Ultramicrotome (PowerTome, San Carlos, CA, USA) using a diamond knife and recovered to 200 mesh Formvar Ni grids, followed by 2% uranyl acetate and saturated lead citrate solution. Visualization was performed at 80 kV in a JEOL JEM 1011 microscope (Akishima, Tokyo, Japan) and digital images were acquired using a Gatan CCD digital camera (Little Egg Harbor Township, NJ, USA).

### 3.5. Statistical Analysis

Analysis of variance (ANOVA) (one and two way) was performed, followed by Tukey’s test and a Bonferroni post hoc test for multiple comparisons using the software GraphPad Prism 6.0 (San Diego, CA, USA). Data were presented as the means ± SD of at least three independent experiments. The significance level was defined as * *p* < 0.005, ** *p* < 0.01, *** *p* < 0.001 and **** *p* < 0.0001.

## 4. Conclusions

The use of lycopene in a self-emulsifying drug delivery system demonstrates the following properties, which can be exploited for skin health benefits: (1) it does not present cytotoxicity in cells of the HaCaT lineage; (2) it demonstrates antioxidant capacity; (3) it prevents degradation and has antioxidant activity of DNA; (4) it inhibits enzymes (tyrosinase and elastase) involved in the skin aging process; (5) it suggests an anti-inflammatory effect only for TNF-α, and pro-inflammatory for IL-6 and IL-8; (6) it affects morphological alterations, mainly of the endoplasmic reticulum, resulting from stress from action or stimulus, evident in the free lycopene from red guava, and these alterations do not occur when lycopene is bound in the nanoemulsion.

## Figures and Tables

**Table 1:** Characterization of nanoLPG.

Sample/Diluent	HD (nm)	PdI	ZP (mV)
NanoLPG/Water	205.73 ± 0.31	0.21 ± 0.01	−20.57 ± 0.31

HD: hydrodynamic diameter; PdI: polydispersity index; ZP: zeta potential.

**Table 2:** Antioxidant activity by oxygen radical absorbance capacity (ORAC). Results in μmol TE (Trolox equivalent)/mL.

NanoLPG	LPG
2924.68 ± 234.80	515.14 ± 55.30

**Table 3:** Enzyme relative inhibition of free LPG and nanoLPG at a concentration of 5 µg/mL.

Samples (5 µg/mL)	Enzyme Relative Inhibition (%)	
	Neutrophil Elastase	Tyrosinase
Inhibitor control	99.41 ± 0.005	95.98 ± 0.04
NanoLPG	16.61 ± 1.5	13.82 ± 1.6
LPG	9.61 ± 3.3	11.38 ± 0.8

## Data Availability

The data presented in this study are available on request from the corresponding author.

## Sample Availability

Samples of the compounds are not available from the authors.

## References

[1] Teixeira N., Melo J.C., Batista L.F., Paula-Souza J., Fronza P., Brandão M.G. (2019). Edible fruits from Brazilian biodiversity: A review on their sensorial characteristics versus bioactivity as tool to select research. Food Res. Int..

[2] Borges P.R.S., Edelenbos M., Larsen E., Hernandes T., Nunes E.E., de Barros Vilas Boas E.V., Pires C.R.F. (2022). The bioactive constituents and antioxidant activities of ten selected Brazilian Cerrado fruits. Food Chem. X.

[3] Chiari-Andréo B.G., de Almeida-Cincotto M.G.J., Oshiro J.A., Taniguchi C.Y.Y., Chiavacci L.A., Isaac V.L.B., Grumezescu A.M. (2019). Nanoparticles for cosmetic use and its application. Nanoparticles in Pharmacotherapy.

[4] Rehman A., Tong Q., Jafari S.M., Assadpour E., Shehzad Q., Aadil R.M., Iqbal M.W., Rashed M.M.A., Mushtaq B.S., Ashraf W. (2020). Carotenoid-loaded nanocarriers: A comprehensive review. Adv. Col. Inter. Sci..

[5] Chuo S.C., Sepatar H.M., Setapsr S.H.M., Ahmad A., Jawaid M. (2022). Application of nanotechnology for development of cosmetics. Nanotechnology for the Preparation of Cosmetis Using Plant-Based Extracts.

[6] Yuan L., Pan M., Shi K., Hu D., Li Y., Chen Y., Qian Z. (2022). Nanocarriers for promoting skin delivery of therapeutic agentes. Appl. Mater. Today.

[7] Offords E.A., Gautier J.-C., Avanti O., Scaletta C., Runge F., Krämer K., Applegate L.A. (2002). Photoprotective potential of lycopeme, β-carotne, vitamin E, vitamin C and carnosic acid in uva-irradiated humana skin fibroblastos. Free Radic. Biol. Med..

[8] Andrades E.O., Costa J.M.A.R., Lima Neto F.E.M., Araujo A.R., Ribeiro F.O.S., Vasconcelos A.G., Oliveira A.C.J., Soares Sobrinho J.L., Almeida M.P., Carvalho A.P. (2021). Acetylated cashew gum and fucan for incorporation of lycopene rich extract from red guava (*Psidium guajava* L.) in nanostructured systems: Antioxidant and antitumor capacity. Int. J. Biol. Macromol..

[9] Phan-Thi H., Waché Y. (2014). Isomerization and increase in the antioxidant properties of lycopene from *Momordica cochinchinensis* (gac) by moderate heat treatment with UV Vis spectra as a marker. Food Chem..

[10] Fam V.W., Charoenwoodhipong P., Sivamani R., Holt R.R., Keen C.L., Hackman R.M. (2022). Plant-based foods for skin health: A narrative review. J. Acad. Nutr. Diet..

[11] Naseer S., Hussain S., Naeem N., Pervaiz M., Rahman M. (2018). The phytochemistry and medicinal value of *Psidium guajava* (guava). Clin. Phytosci..

[12] Caseiro M., Ascenso A., Costa A., Creagh-Flynn J., Johnson M., Simões S. (2020). Lycopene in human health. LWT Food Sci. Technol..

[13] Amorim A.G., Souza J., Oliveira A., Santos R., Vasconcelos A., Souza L., Araujo T., Cabral W., Silva M., Mafud A. (2020). Anti-inflammatory and antioxidant activity improvement of lycopene from guava on nanoemulsifying system. J. Dispers. Sci. Technol..

[14] Moia V.M., Portilho F.L., Pádua T.A., Corrêa L.B., Ricci-Junior E., Rosas E.C., Alencar L.M.R., Sinfronio F.S.M., Sampson A., Iram S.H. (2020). Lycopene used as Anti-inflammatory Nanodrug for the Treatment of Rheumathoid Arthritis: Animal assay, Pharmacokinetics, ABC Transporter and Tissue Deposition. Colloids Surf. B Biointerfaces.

[15] Amorim A.G.N., Souza J.M.T., Santos R.C., Gullón B., Oliveira A., Santos L.F.A., Virgino A.L.E., Mafud A.C., Petrilli H.M., Mascarenhas Y.P. (2018). HPLC-DAD, ESI–MS/MS, and NMR of lycopene isolated from *P. guajava* L. and its biotechnological applications. Eur. J. Lipid Sci. Technol..

[16] Vasconcelos A.G., Amorim A.G.N., Dos Santos R.C., Souza J.M.T., de Souza L.K.M., Araújo T.S.L., Nicolau L.A.D., Carvalho L.L., Aquino P.E.A., Martins C.S. (2017). Lycopene rich extract from red guava (*Psidium guajava* L.) displays anti-inflammatory and antioxidant profile by reducing suggestive hallmarks of acute inflammatory response in mice. Food Res. Int..

[17] Vasconcelos A.G., Barros A.L.A.N., Cabral W.F., Moreira D.C., Silva I.G.M., Silva-Carvalho A.E., Almeida M.P., Albuquerque L.F.F., Santos R.C., Brito A.K.S. (2021). Promising self-emulsifying drug delivery system loaded with lycopene from red guava (*Psidium guajava* L.): In vivo toxicity, biodistribution and cytotoxicity on DU-145 prostate cancer cells. Cancer Nanotechnol..

[18] How C.W., Abdullah R., Abbasalipourkabir R. (2011). Physicochemical properties of nanostructured lipid carriers as colloidal carrier system stabilized with polysorbate 20 and polysorbate 80. Afr. J. Biotechnol..

[19] Vasconcelos A.G., Valim M.O., Amorim A.G.N., do Amaral C.P., Almeida M.P., Borges T.K.S., Socodato R., Portugal C.C., Brand G.D., Matto J.S.C. (2020). Cytotoxic activity of poly-caprolactone lipid-core nanocapsules loaded with lycopene-rich extract from red guava (*Psidium guajava* L.) on breast cancer cell. Food Res. Int..

[20] Santos P.P., Paese K., Guterres S.S., Pohlmann A.R., Costa T.H., Jablonski A., Flôres S.H., Rios A.O. (2015). Development of lycopene-loaded lipid-core nanocapsules: Physicochemical characterization and stability study. J. Nanoparticle Res..

[21] Pal V.K. (2011). Self Emulsifying Drug Delivery System. J. Pharm. Res. Opin..

[22] Baranowska-Wójcik E., Szwajgier D., Oleszczuk P., Winiarska-Mieczan A. (2020). Efects of titanium dioxide nano particles exposure on human health—A review. Biol. Trace Elem. Res..

[23] Wang C., Guan W., Chen R., Levi-Kalisman Y., Xu Y., Zhang L., Zhou M., Xu G., Dou H. (2020). Fluorescent glycan nanoparticle based FACS assays for the identifcation of genuine drug-resistant cancer cells with diferentiation potential. Nano Res..

[24] Silva B., Marto J., São Braz B., Delgado E., Almeida A.J., Gonçalves L. (2020). New nanoparticles for topical ocular delivery of erythropoietin. Int. J. Pharm..

[25] Smith M.C., Crist R.M., Clogston J.D., McNeil S.E. (2017). Zeta potential: A case study of cationic, anionic, and neutral liposomes. Anal. Bioanal. Chem..

[26] Ha T.V.A., Kim S., Choi Y., Kwak H.-S., Lee S.J., We J., Oey I., Ko S. (2015). Antioxidant activity and bioaccessibility of size-different nanoemulsions for lycopene-enriched tomato extract. Food Chem..

[27] Campos K.K.D., Araújo G.R., Martins T.L., Bandeira A.C.B., Costa G.P., Talvani A., Garcia C.C.M., Oliveira L.A.M., Costa D.C., Bezerra F.S. (2017). The antioxidant and anti-inflammatory properties of lycopene in mice lungs exposed to cigarette smoke. J. Nutr. Biochem..

[28] Wen W., Chen X., Huang Z., Chen D., Yu B., He J., Luo Y., Yan H., Chen H., Zheng P. (2022). Dietary lycopene supplementation improves meat quality, antioxidant capacity and skeletal muscle fiber type transformation in finishing pigs. Anim. Nutr..

[29] Bonifácio-Lopes T., Vila Boas A.A., Coscueta E.R., Costa E.M., Silva S., Campos D., Teixeira J.A., Pintado M. (2020). Bioactive extracts from brewer’s spent grain. Food Funct..

[30] Nurrochmad A., Wirasti W., Dirman A., Lukitaningsih E., Rahmawati A., Fakhrudin N. (2018). Effects of antioxidant, anti-collagenase, anti-elastase, anti-tyrosinase of the extract and fraction from Turbinaria decurrens Bory. Indones. J. Pharm..

[31] Shirzad M., Hamedi J., Motevaseli E., Modarressi M.H. (2018). Anti-elastase and anti-collagenase potential of Lactobacilli exopolysaccharides on human fibroblast. Artif. Cells Nanomed. Biotechnol..

[32] Cunha S.A., Pintado M.E. (2021). Bioactive peptides derived from marine sources: Biological and functional properties. Trends Food Sci. Technol..

[33] Kim S.Y., Kwon Y.M., Kim K.W., Kim J.Y.H. (2021). Exploring the Potential of Nannochloropsis sp. Extract for Cosmeceutical Applications. Mar. Drugs.

[34] Lee S.-M., Lee Y.-R., Cho K.-S., Cho Y.-N., Lee H.A., Hwang D.-Y., Jung Y.-J., Son H.-Y. (2015). Stalked sea squirt (*Styela clava*) tunic waste as a valuable bioresource: Cosmetic and antioxidant activities. Process Biochem..

[35] Ambarwati N.S.S., Armandari M.O., Widavat W., Desmiaty Y., Elva B., Arifianti A.E., Ahmad I. (2022). In vitro studies on the cytotoxicity, elastase, and tyrosinase inhibitory activities of tomato (*Solanum lycopersicum* Mill.) extract. J. Adv. Pharm. Technol. Res..

[36] Guerra A.S., Hoyos C.G., Molina-Ramirez C., Velásquez-Cock J., Vélez L., Gañan P., Eceiza A., Goff H.D., Zuluaga R. (2021). Extraction and preservation of lycopene: A review of the advancements offered by the value chain of nanotechnology. Trends Food Sci. Technol..

[37] Komijani M., Mohebbi M., Ghorani B. (2022). Assembly of electrospun tri-layered nanofibrous structure of zein/basil seed gum/zein for increasing the bioaccessibility of lycopene. LWT Food Sci. Technol..

[38] Falsafi S.R., Rostamabadi H., Babazadeh A., Tarhan O., Rashidinejad A., Boostani S., Khoshnoudi-Nia S., Akabari-Alavijeh S., Shaddel R., Jafari S.M. (2022). Lycopene nanodelivery systems; recent advances. Trends Food Sci. Technol..

[39] Fakhri V., Jafari A., Shafiei M.A., Ehteshamfar M.V., Khalighiyan S., Hosseini H., Goodarzi V., Wurm F.R., Moghaddam M.M., Khonakdar H.A. (2021). Development of physical, mechanical, antibacterial and cell growth properties of poly(glycerol sebacate urethane) (PGSU) with helping of curcumin and hydroxyapatite nanoparticles. Polym. Chem..

[40] Monika P., Chandraprabha M.N., Murthy K.N.C., Rangarajan A., Waiker P.V., Sathish M. (2022). Human primary chronic wound derived fibroblasts demonstrate differential pattern in expression of fibroblast specific markers, cell cycle arrest and reduced proliferation. Exp. Mol. Pathol..

[41] Hao Y., Zhao W., Zhang H., Zheng W., Zhou Q. (2022). Carboxymethyl chitosan-based hydrogels containing fibroblast growth factors for triggering diabetic wound healing. Carbohydr. Polym..

[42] Zheng Z., Yin Y., Lu R., Jiang Z. (2019). Lycopene ameliorated oxidative stress and inflammation in type 2 diabetic rats. J. Food Sci..

[43] MacManus C.F., Pettigrew J., Seaton A., Wilson C., Maxwell P.J., Berlingeri S., Purcell C., McGurk M., Johnston P.G., Waugh D.J.J. (2007). Interleukin-8 Signaling Promotes Translational Regulation of Cyclin D in Androgen-Independent Prostate Cancer Cells. Mol. Cancer Res..

[44] Wang Y., Xu R.C., Zhang X.L., Niu X.L., Qu Y., Li L.Z., Meng X.Y. (2012). Interleukin-8 secretion by ovarian cancer cells increases anchorage-independent growth, proliferation, angiogenic potential, adhesion and invasion. Cytokine.

[45] Cui G., Li G., Pang Z., Florholmen J., Goll R. (2022). The presentation and regulation of the IL-8 network in the epithelial cancer stem-like cell niche in patients with colorectal cancer. Biomed. Pharmacother..

[46] Tang S.-C., Liao P.-Y., Hung S.-J., Ge J.-S., Chen S.M., Lai J.-C., Hsiao Y.-P., Yang J.-H. (2017). Topical application of glycolic acid suppresses the UVB induced IL-6, IL-8, MCP-1 and COX-2 inflammation by modulating NF-kB signaling pathway in keratinocytes and mice skin. J. Dermatol. Sci..

[47] Machado M., Costa E.M., Silva S., Rodriguez-Alcalá L.M., Gomes A.M., Pintado M. (2022). Pomegranate Oil’s Potential as an Anti-Obesity Ingredient. Molecules.

[48] Pisani S., Dorati R., Genta I., Benazzo M., Conti B., Mello A.P. (2021). A study focused on macrophages modulation induced by the Polymeric Electrospun Matrices (EL-Ms) for application in tissue regeneration: In vitro proof of concept. Int. J. Pharm..

[49] Matjaž M.J., Skarabot M., Gasperlin M., Jankovic B. (2019). Lamellar liquid crystals maintain keratinocytes′ membrane fluidity: An AFM qualitative and quantitative study. Int. J. Pharm..

[50] Rocha M.C.O., Silva P.B., Radicchi M.A., Andrade B.Y.G., Oliveira J.V., Venus T., Merker C., Estrela-Lopis I., Longo J.P.F., Báo S.N. (2020). Docetaxel-loaded solid lipid nanoparticles prevent tumor growth and lung metastasis of 4T1 murine mammary carcinoma cells. J. Nanobiotech..

[51] Paiva K.L.R., Radicchi M.A., Báo S.N. (2022). In Vitro Evaluation of NLS-DTX Activity in Triple-Negative Breast Cancer. Molecules.

[52] Strus P., Borensztejn K., Szczepankiewicz A.A., Lisiecki K., Czarnocki Z., Nieznanska H., Wojcik C., Bialy L.P., Mlynarczuk-Bialy I. (2021). Novel podophyllotoxin and benzothiazole derivative induces transitional morphological and functional changes in HaCaT cells. Toxicol. In Vitro.

[53] Cervellati F., Benedusi M., Manarini F., Woodby B., Russo M., Valacchi G., Pietrogrande M.C. (2020). Proinflammatory properties and oxidative effects of atmospheric particle components in human keratinocytes. Chemosphere.

[54] Yen C.C., Chang C.-W., Hsu M.-C., Wu Y.-T. (2017). Self-nanoemulsifying drug delivery system for resveratrol: Enhanced oral bioavailability and reduced physical fatigue in rats. Int. J. Mol. Sci..

[55] Silva S., Costa E.M., Vicente S., Veiga M., Calhau C., Morais R.M., Pintado M.E. (2017). DNA agarose gel electrophoresis for antioxidant analysis: Development of a quantitative approach for phenolic extracts. Food Chem..

